# Rapid flotation of *Microcystis wesenbergii* mediated by high light exposure: implications for surface scum formation and cyanobacterial species succession

**DOI:** 10.3389/fpls.2024.1367680

**Published:** 2024-04-03

**Authors:** Tiantian Yang, Jiaxin Pan, Huaming Wu, Cuicui Tian, Chunbo Wang, Bangding Xiao, Min Pan, Xingqiang Wu

**Affiliations:** ^1^ Key Laboratory of Algal Biology of Chinese Academy of Sciences, Institute of Hydrobiology, Chinese Academy of Sciences, Wuhan, China; ^2^ Kunming Dianchi & Plateau Lakes Institute, Dianchi Lake Ecosystem Observation and Research Station of Yunnan Province, Kunming, China; ^3^ College of Hydraulic and Envrionmental Engineering, China Three Gorges University, Yichang, China; ^4^ Institute for Environmental Sciences, University of Koblenz-Landau, Landau, Germany

**Keywords:** *Microcystis*, surface scum formation, cell aggregation, micro-bubbles, extracellular polymeric substance

## Abstract

Increasing occurrences of *Microcystis* surface scum have been observed in the context of global climate change and the increase in anthropogenic pollution, causing deteriorating water quality in aquatic ecosystems. Previous studies on scum formation mainly focus on the buoyancy-driven floating process of larger *Microcystis* colonies, neglecting other potential mechanisms. To study the non-buoyancy-driven rapid flotation of *Microcystis*, we here investigate the floating processes of two strains of single-cell species (*Microcystis aeruginosa* and *Microcystis wesenbergii*), which are typically buoyant, under light conditions (150 μmol photons s^−1^ m^−2^). Our results showed that *M. wesenbergii* exhibited fast upward migration and formed surface scum within 4 hours, while *M. aeruginosa* did not form visible scum throughout the experiments. To further explore the underlying mechanism of these processes, we compared the dissolved oxygen (DO), extracellular polymeric substance (EPS) content, and colony size of *Microcystis* in different treatments. We found supersaturated DO and the formation of micro-bubbles (50–200 µm in diameter) in *M. wesenbergii* treatments. *M. aeruginosa* produces bubbles in small quantities and small sizes. Additionally, *M. wesenbergii* produced more EPS and tended to aggregate into larger colonies. *M. wesenbergii* had much more derived-soluble extracellular proteins and polysaccharides compared to *M. aeruginosa*. At the same time, *M. wesenbergii* contains abundant functional groups, which was beneficial to the formation of agglomerates. The surface scum observed in *M. wesenbergii* is likely due to micro-bubbles attaching to the surface of cell aggregates or becoming trapped within the colony. Our study reveals a species-specific mechanism for the rapid floatation of *Microcystis*, providing novel insights into surface scum formation as well as succession of cyanobacterial species.

## Graphical Abstract

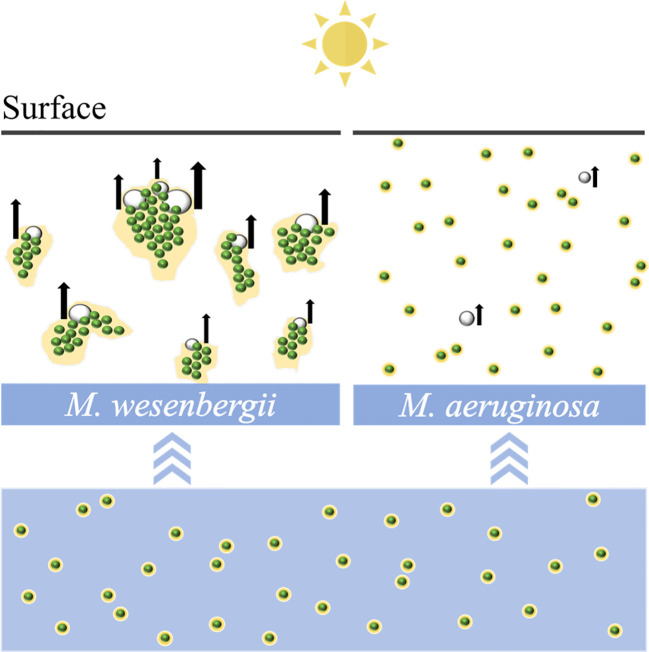

## Highlights

• EPS produced by *Microcystis wesenbergii* can contribute to the formation of large aggregates.
• The formation of aggregate and micro-bubble can drive the surface scum formation.
• Light and EPS contributed to the formation of the large algal aggregate.

## 1 Introduction

Cyanobacterial blooms exist in many freshwater bodies worldwide (Schindler, 1974). *Microcystis* spp., which can form colonies ranging in size from a few microns to a few millimeters, are the most common and ubiquitous toxic blooms (Paerl et al., 2014). Under warming and eutrophic conditions, *Microcystis* cells have the propensity to aggregate and float upward, giving rise to harmful *Microcystis* blooms (Luerling et al., 2017; Zhu et al., 2023). The extensive proliferation of harmful *Microcystis* has various negative effects on human health and environmental safety (Fiehn et al., 1998; Susanna et al., 2020). This phenomenon results in the depletion of dissolved oxygen in the water, causing disruptions to aquatic ecosystems (Paerl et al., 2011; Guo et al., 2022), in which the decay of biomass ultimately leads to oxygen depletion, causing a complete alteration of the aquatic environment (Hallegraeff, 1993). Microcystins are toxins produced by a variety of bloom-forming cyanobacteria that can cause hepatotoxicity in humans and animals (Zhou et al., 2021).

The formation of *Microcystis* blooms is affected by various biotic and abiotic factors, including nutrients, light, temperature (Andrew, 1991; Soranno, 1997; Hans and Valerie, 2012; Min et al., 2012), hydrodynamic conditions (Medrano et al., 2013; Chao et al., 2017), predation (Wang et al., 2010), and buoyancy of *Microcystis* colonies (Jacco and Luuc, 1984; Medrano et al., 2016). Due to the buoyancy regulation, *Microcystis* is capable of forming surface scum in eutrophic lakes (Chorus, 1999; Rainer et al., 2003). Many former studies suggest that buoyancy provides several competitive advantages for *Microcystis* over phytoplankton, including the capability to acquire light and carbon dioxide at the uppermost layer, and grazing avoidance (Reynolds and Walsby, 1975; Lovelock et al., 2008). *Microcystis* had a diurnal migration pattern, and migration causes were affected by multiple factors (Wu et al., 2019). This development will be rapid, probably on a characteristic timescale of approximately a day (Timothy et al., 2009). The sudden increases in biomass at the surface layer may lead to long-term proliferative cell interactions and mass migration of biomass. In many cases, this migration ends up forming thick scum on the surface of the water (Anne et al., 2016).

There is currently research evidence demonstrating that the regulation of gas buoyancy on vesicles and carbohydrate ballast is a crucial factor in the migration of *Microcystis* colony (Colin et al., 2002). The hypothesis posited suggests that the irreversible buoyancy of cyanobacterial colonies is induced by the growth of gas bubbles on or within the mucilage of the colonies (Medrano et al., 2016). They hypothesized that the irreversible buoyancy of cyanobacterial colonies is induced by the growth of bubbles on or inside the colony mucilage. These bubbles grow under conditions of oxygen supersaturation. Meanwhile, many abiotic factors can affect the buoyancy of *Microcystis* through these mechanisms (Hans and Jef, 2008; Paerl and Otten, 2013). For instance, light has been found to regulate buoyancy through the carbohydrate ballast mechanism. Specifically, *Microcystis* loses buoyancy under high light conditions, while it regains buoyancy under low light conditions. This allows *Microcystis* to exhibit a diel migration pattern, where it floats upward to the surface at night and sinks during the daytime. Although this migration pattern has been confirmed by many lake studies (Ma et al., 2015), there are exceptions where *Microcystis* forms scum on a shorter timescale of hours and can persist at the water surface even under high light conditions during the daytime. This may imply the existence of additional mechanisms for the rapid *Microcystis* flotation and the surface scum formation under strong light conditions.

To fill the knowledge gaps, the influence of high light on the floatation and surface scum formation of *Microcystis* was investigated in this study. We used two different strains of *Microcystis* species (*Microcystis wesenbergii* and *Microcystis aeruginosa*), which are neutrally buoyant, to study the non-buoyancy-driven floatation of *Microcystis* with laboratory experiments. We hypothesize that the bubbles generated from photosynthesis during high light exposure can felicitate the rapid floatation of *Microcystis*. We aim to study the mechanism of rapid floatation of *Microcystis* driven by bubble formation. This study is expected to provide new implications for the mechanism for the formation of *Microcystis* blooms as well as cyanobacterial species succession.

## 2 Materials and methods

### 2.1 *Microcystis* strains and culture conditions

Two different *Microcystis* strains (*M. wesenbergii*, FACHB-908, and *M. aeruginosa*, FACHB-905) used in this study were generously provided by the Freshwater Algae Culture Collection at the Institute of Hydrobiology, Chinese Academy of Sciences (FACHB-collection, Wuhan, China). The strains were cultured in BG11 medium (**Supplementary Table 1**) at 25°C with a 16-hour light/8-hour dark cycle of 32 µmol photons s^−1^ m^−2^ to obtain a cell density of ca. 800 μg/L (Lin et al., 2017).

### 2.2 Experimental design

To study the effect of light intensity on the *Microcystis* aggregation and their upward floating to the surface, *M. aeruginosa* and *M. wesenbergii* were diluted to the same initial Chl*a* concentration (ca. 800 μg/L). Two types of *Microcystis* were placed in separate 50-mL glass tubes (height, 20 cm) under strong light (150 μmol photons s^−1^ m^−2^) and dark conditions. The light source was derived from a LED lamp located on the side of the test tubes to provide light. Dark conditions were carried out in a closed cabinet. The room temperature was maintained at 25°C. Samples that float to the surface were mainly collected to measure the dissolved oxygen, electrolytic potential, and extracellular polysaccharides. All the treatments and controls were performed in triplicate. A Canon camera was used to capture images of *Microcystis* aggregation and floating to determine the state and size of the aggregation under light conditions.

### 2.3 Measurement and characterization of algal aggregate extracellular polymeric substances derived from *Microcystis*

To analyze the composition of special substances in extracellular polymeric substances (EPS) released by two different strains of *Microcystis* under light conditions, two treatments and two controls were prepared, as follows. To prepare surface aggregate samples, 50 mL of algal solution containing *M. wesenbergii* (A) and *M. aeruginosa* (B) was used. Light conditions were used as treatment and dark conditions as control, with the same density of *Microcystis* solution (L and D as abbreviations for light and dark, respectively). The samples were placed in a 50-mL centrifuge tube. Three parallel samples were analyzed for each group using the method described below.

#### 2.3.1 EPS extraction and quantification

The extracellular polymeric substances were collected from the *M. aeruginosa* and *M. wesenbergii* cultures (stationary phase) according to the method of Xiao et al. (2019). Algae suspensions (10 mL) were centrifuged at 11,000 *g* and 4°C for 15 minutes, separating the supernatant and algal cells. The EPS fractions were then divided into soluble EPS (S-EPS) and bound EPS (B-EPS). The supernatant was used to determine the soluble EPS. The pH was adjusted to 10 using the 1 mol/L of sodium hydroxide. The samples were then placed in a water bath sonicator at intermediate power (25 kHz) and 45°C to separate cells from loosely bound EPS. The resulting EPS were classified as conjunction type and stored at −20°C until analysis. The total EPS content in the algal aggregate was calculated as the sum of polysaccharides and proteins. The protein content was determined using Coomassie brilliant blue (Marion, 1976). The polysaccharides were analyzed using the phenol-sulfuric acid method (Dubois et al., 1951).

#### 2.3.2 EPS fluorescence staining and confocal laser scanning microscopy analysis

A modified fluorescence staining method according to Liu (Liu et al., 2020) was used to observe the components of EPS. The *Microcystis* samples were collected from the surface layer and washed three times with phosphate buffer (pH = 7) to remove the medium. They were then fixed with 2.5% glutaraldehyde. The SYTO63 stain (Thermo Fisher Scientific, Waltham, MA, USA), fluorescein isothiocyanate (FITC), and calcofluor white were used to stain bacterial cells, proteins, and polysaccharides, respectively. The spatial distribution of various components in EPS was observed using confocal laser scanning microscopy (CLSM) (TCS, Leica, Wetzlar, Germany). The excitation wavelengths for polysaccharides and proteins were 400 nm and 480 nm, with the emission wavelengths of 480 nm and 550 nm, respectively (Chen et al., 2007). After each staining procedure, samples were washed at least twice with phosphate-buffered saline (PBS) (pH = 7.2) to remove excess stains. The polysaccharides are represented by blue fluorescence, the proteins are represented by green fluorescence, and the bacteria are represented by red fluorescence (Badiaa et al., 2010). The sample preparation process should be carried out in a darkroom to avoid fluorescence.

#### 2.3.3 Fourier transform infrared spectrum and fluorescence spectrometer analysis

The Fourier transform infrared (FT-IR) spectrum of EPS samples was analyzed using a Fourier transform infrared spectrometer (Nicolet 6700, Thermo Scientific Co., Ltd., USA). All samples were washed twice with PBS (pH = 7.2), then lyophilized, and stored at −20°C (Bo et al., 1996). Before FT-IR scanning, samples were ground with IR-grade KBr powder and molded into a disc. The infrared absorption spectra of transmittance or absorbance with wave number or wavelength were obtained by Fourier transform. The components of organic chemicals were analyzed using sub-peak spectra obtained from the original spectra through curve fitting.

The fluorescence intensity of the protein-like components and humic acid-like components in samples was measured using the fluorescence excitation–emission matrix (3D EEM) with a fluorescence spectrometer (Hitachi F4700, Hitachi, Tokyo, Japan) (Yunlin et al., 2014). The slit width was set to 5 nm, and the photomultiplier was set to a voltage of 720 V. The excitation scanning range was 250–450 nm, and the emission scanning range was 300–550 nm and 2 nm. The EEM data of deionized water were also subtracted to remove the effect of Raman scattering (Markus et al., 2010).

### 2.4 The *Microcystis* algae cell density

The cell density was counted three times in a hemocytometer using an optical microscope (BX43, Olympus Corporation, Tokyo, Japan) at ×40 magnification.

### 2.5 Statistical analysis

Variance analysis (ANOVA) was used to determine the difference in EPS content released from different strains of *Microcystis*. Statistical significance was set at *p* < 0.05. All significant differences between samples were determined using SPSS version 25 (IBM, USA). Graphs were generated using Origin 8.0 software (OriginLab, Northampton, MA, USA).

## 3 Results

### 3.1 Effect of light intensity on the surface *Microcystis* aggregates

As shown in **Supplementary Video 1**, *M. wesenbergii* suddenly produced bubbles under strong light intensity in the middle stage, causing the algal biomass to rise and reach a layer of foam at the air–water interface. Under suitable nutrient and light conditions, the dissolved oxygen (DO) in photosynthetic active cells was supersaturated to form bubbles. Bubbles were wrapped and expanded until buoyancy was sufficient to pull *M. wesenbergii* aggregates to the water surface and to form a stable surface bloom after 4 hours. However, this phenomenon was not observed in *M. aeruginosa*. *M. aeruginosa* only produced very few bubbles uniformly distributed in the water column.

**Figure 1:**
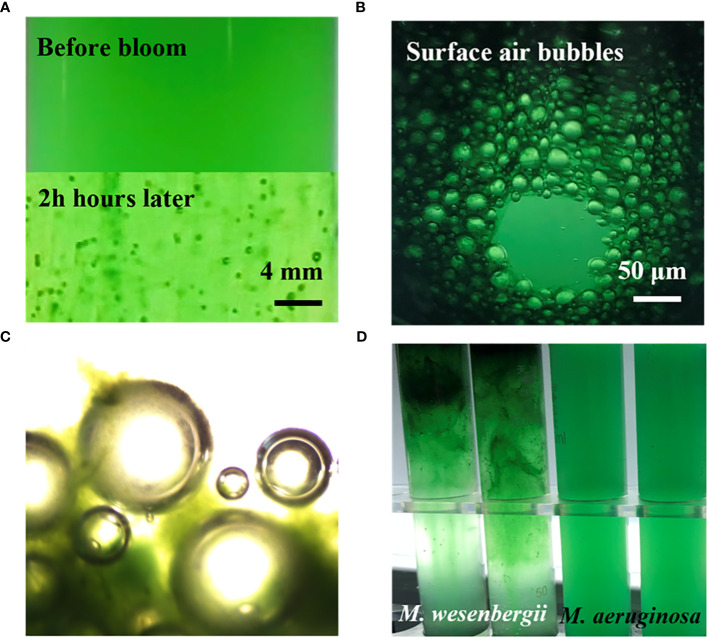
Contrast before and after the formation of cyanobacterial blooms. **(A)** Bubbles of *Microcystis wesenbergii* forming during experimentation. **(B)** Aggregation of the biomass and bubble production of *M. wesenbergii* during a bloom. **(C)** Microscopic bubbles and *Microcystis* aggregates. **(D)** Different experimental phenomena produced by *M. wesenbergii* and *Microcystis aeruginosa*.

**Figure 1** illustrates the typical time evolution of the system. The culture kept in darkness remained homogeneous throughout the experiment. We observed a massive migration of the biomass toward the water surface in the sample exposed to high light exposure. The floating phenomena of *M. wesenbergii* and *M. aeruginosa* were distinct (**Figure 1D**). The migration and aggregation of *M. wesenbergii* resulted from the generation and floating migration of bubbles. At the same time, the bubbles cause *M. wesenbergii* to rise to the surface (**Figure 1C**). Approximately 1 hour after the start of the experiment, bubbles began to form gradually. For approximately 2 hours, the test tubes were filled with numerous stable bubbles (**Figure 1A**). When the test tubes were slightly shaken, the bubbles floated up quickly and did not adhere to the wall of the test tubes. This indicates that the *M. wesenbergii* is covering them, and these bubbles continued to increase until they were sufficiently supported to float. It should be noted that cyanobacteria under light conditions persist in the bacterial foam formed at the surface (**Figure 1C**) and did not sink even after 1 week. In contrast, the samples placed in darkness settled. Additionally, we observed supersaturated DO and the formation of micro-bubbles (50–200 μm in diameter) in *M. wesenbergii* treatments, while *M. aeruginosa* produced bubbles in small quantities and small sizes (**Supplementary Figure 1**).

### 3.2 Changes in physicochemical indexes of surface *Microcystis* aggregates (DO and zeta potential)

**Figure 2:**
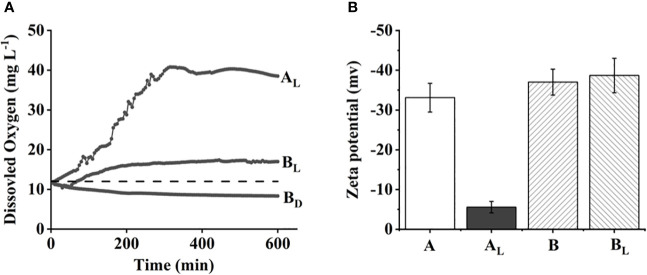
Surface dissolved oxygen (DO) concentration over time **(A)** and zeta potential of two different strains of *Microcystis* **(B)**. The dashed line in panel A indicates equilibrium value with air.

When the bloom in *M. wesenbergii* produced a large number of bubbles, we hypothesized that the gas was oxygen, a by-product of photosynthesis. We then measured the dynamic DO contents at the air–water interface under dark and light conditions. As shown in **Figure 2A**, the DO concentration of the two different strains of *Microcystis* was approximately 10.8 mg/L initially. The dissolved oxygen concentration of *M. wesenbergii* increased linearly at a rate of 5 mg L^−1^ h^−1^ over the next 4 hours under light conditions. Under dark conditions, the DO concentration decreased steadily and eventually fell below the equilibrium value of 8.1 mg/L, indicating active aerobic respiration in the system. In contrast, under light conditions, the DO concentration of *M. wesenbergii* increased linearly at a rate of 2 mg L^−1^ h^−1^ until the detection limit of the DO probe (42 mg/L) approximately 300 minutes after the start of the experiment. The rate of DO increase for *M. aeruginosa* was much lower. This was consistent with the experimental phenomenon (**Figure 1D**).

Zeta potential is usually used to monitor the electrostatic neutralization of particles to explain the relationship between particle instability and floc formation (Arya et al., 2019). The magnitude of the electrokinetic potential is associated with the stability of the solution. At the start of the experiment, the zeta potentials of *M. aeruginosa* and *M. wesenbergii* solution were similar below 30 mV. However, by the end of the experiment, the zeta potentials of the *M. wesenbergii* solution had significantly increased to a lower value of (−5 mV). The electric potential of *M. wesenbergii* changed noticeably before and after the experiment, indicating the neutralization of electrostatic particles by static electricity. This phenomenon was not observed in the other treatment groups, confirming the consistency of the experimental results.

### 3.3 Changes in the content of extracellular material in *Microcystis* aggregates

**Figure 3:**
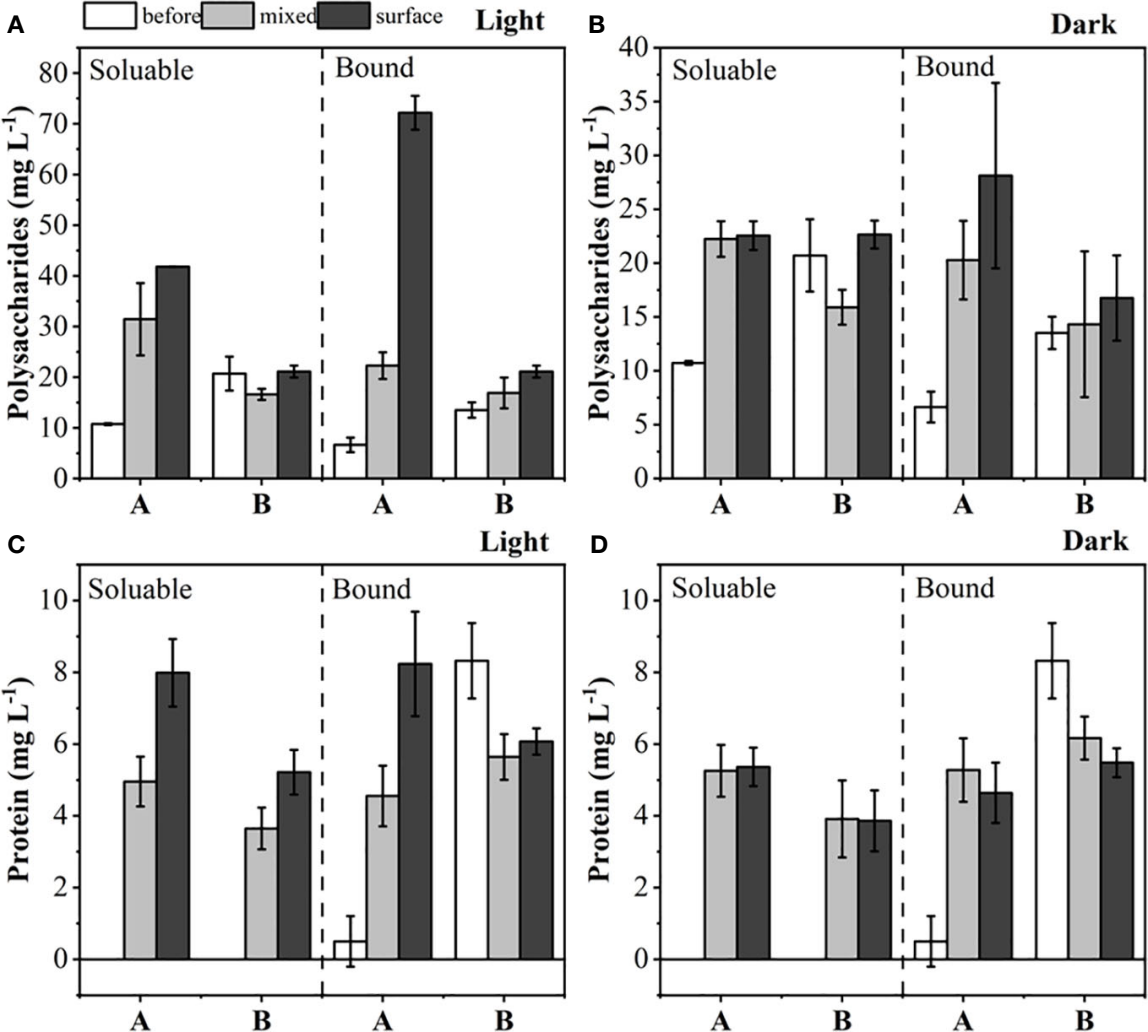
The content of polysaccharides in light **(A)** and darkness **(B)** before and after the experiment, and proteins in light **(C)** and darkness **(D)** before and after the experiment.

Polysaccharides and protein content were measured in mixed and surface samples of *M. wesenbergii* and *M. aeruginosa* under dark and light conditions. **Figure 3** shows that under light conditions, the polysaccharides secreted by *M. wesenbergii* were significantly higher than those secreted by *M. aeruginosa* (*p* < 0.05), but there was little difference under dark conditions. Under light conditions, the polysaccharides secreted by the surface layer of *M. wesenbergii* were significantly higher than those in the water column (*p* < 0.05). Meanwhile, the content of polysaccharides secreted by *M. aeruginosa* on the surface and the water column was basically the same (*p* > 0.05). In the mixed samples of *M. wesenbergii*, the levels of dissolved polysaccharides and bound polysaccharides were 10.7 mg/L and 6.6 mg/L, respectively; the surface samples showed higher levels of polysaccharides, corresponding to 41.8 mg/L and 72.2 mg/L, respectively. The levels of dissolved proteins and loosely bound proteins increased from nearly zero at the beginning of the experiment to approximately 8 mg/L. In contrast, the mixed samples and surface samples of *M. aeruginosa* showed constant levels of polysaccharides and protein under both illuminated and dark conditions. The concentration of dissolved proteins increased from 0 mg/L to approximately 4 mg/L, while the level of bound proteins slightly decreased. Significant differences in the secretion of dissolved polysaccharides and bound polysaccharides were observed between *M. aeruginosa* and *M. wesenbergii* (*p* < 0.01). The polysaccharide content of *M. wesenbergii* decreased significantly under dark conditions, and both illumination and algal species had a significant impact on the concentration of dissolved polysaccharides (*p* < 0.01).

### 3.4 Qualitative analysis of extracellular species in *Microcystis* aggregates

#### 3.4.1 CLSM picture of organic constituents in EPS

**Figure 4:**
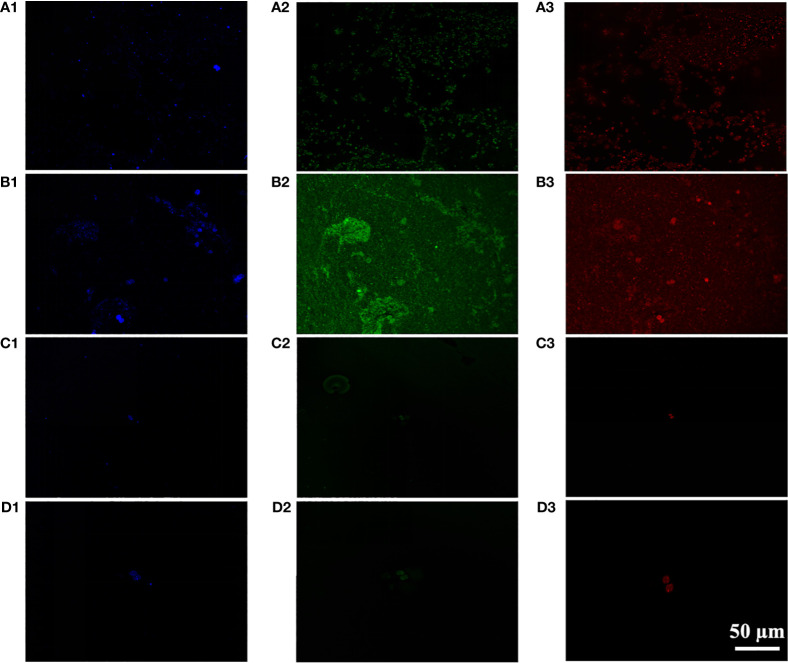
CLSM images of unicellular *Microcystis wesenbergii* before **(A)** after **(B)** and *Microcystis aeruginosa* before **(C)** after **(D)** floating up to the water surface (1, polysaccharides; 2, proteins; 3, bacteria). CLSM, confocal laser scanning microscopy.

CLSM analysis was conducted to investigate the distribution of cells, proteins, and polysaccharides in the *Microcystis* aggregate (**Figure 4**). The results showed a significant increase in polysaccharides and protein of *M. wesenbergii* after illumination (**Figures 4A, B**), while no significant increase was observed in *M. aeruginosa* (**Figures 4C, D**). As shown in **Figure 4**, the membrane formed by the surface layer of *M. wesenbergii* samples has a network or membrane-like structure, which contains obvious polysaccharides and protein components. Additionally, *M. wesenbergii* cells formed large aggregates. As time increased, the protein and polysaccharide contents also increased significantly (**Figure 3**). It was worth noting that the content of both protein and polysaccharides was related to cell density (**Figure 4B**). However, the initial biomass remained consistent, and the protein surrounding the cells of *M. wesenbergii* was denser. This may be closely related to cell distribution and content.

#### 3.4.2 Three-dimensional fluorescence in superficial surface *Microcystis* aggregates

**Figure 5:**
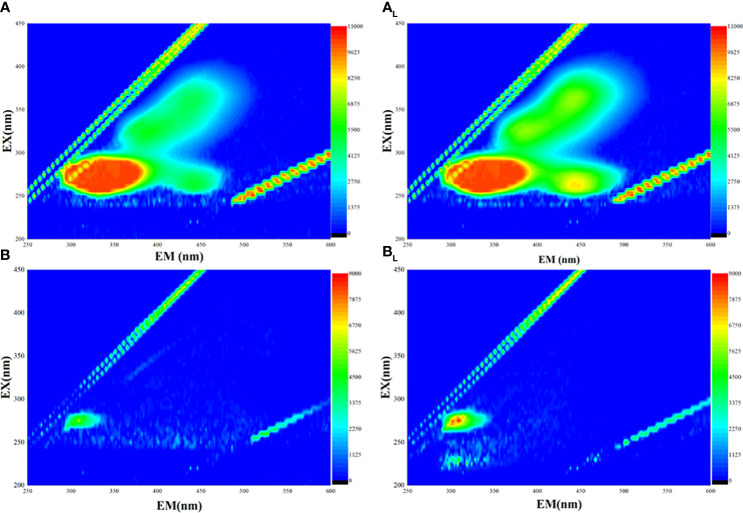
Three-dimensional fluorescence of two different strains of *Microcystis*. **(A)** *Microcystis wesenbergii* before light exposure. (A_L_) *M. wesenbergii* under light. **(B)** *Microcystis aeruginosa* before light exposure. (B_L_) *M. aeruginosa* under light.

The 3D EEM fluorescence spectra revealed three obvious fluorescent peaks in the EPS sample of strain FACHB 908 (**Figure 5**). Two of these peaks, observed at the excitation/emission wavelengths (Ex/Em) of 205/300 and 230/300, were identified as tyrosine protein-like. The third peak, observed at Ex/Em 235/350, was identified as tryptophan protein-like. No fluorescent peak was assigned for the presence of humic acid, suggesting that it may not be present in the EPS of laboratory trains.

#### 3.4.3 FT-IR spectra in superficial surface *Microcystis* aggregates

**Figure 6:**
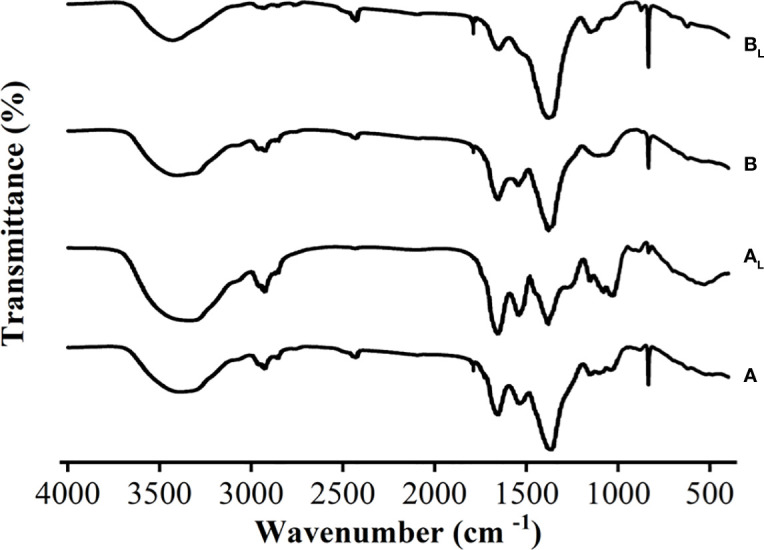
FT-IR spectra of two different strains of *Microcystis* before and after the experiment. **(A)** *Microcystis wesenbergii* before light exposure. (A_L_) *M. wesenbergii* under light. **(B)** *Microcystis aeruginosa* before light exposure. (B_L_) *M. aeruginosa* under light. FT-IR, Fourier transform infrared.

FT-IR analysis was conducted on *M. wesenbergii* and *M. aeruginosa* to compare the composition of specific substances that produce surface blooms after light exposure. The functional groups were used to determine the type of compounds (Jingyun et al., 2010). The FT-IR results showed that there were no significant differences in most of the bands in *M. aeruginosa* before and after the experiment. As shown in **Figure 6**, the peak appeared at 1,726 cm^−1^ after the experiment. At the spectral width of 1,537–1,652 cm^−1^, the intensity of the bound amide group decreased, representing the NH in the peptide bond. The NH bond breaks with the CN bond. In the spectrum of *M. wesenbergii*, the NH stretch vibration at 3,382–3,338 cm^−1^ significantly increased, indicating the full involvement of the NH bond in the process of surface water bloom formation and the creation of a surface film. The broad peak of 3,408–3,431 cm^−1^ in the spectrum of *M. aeruginosa* was stronger than before the experiment, and the intensity of the spectrum of *M. wesenbergii* was stronger. These groups exhibited broad bands of polysaccharides (3,700–3,000 cm^−1^ and 1,500–1,200 cm^−1^) and proteins (1,700–1,600 cm^−1^ and 1,200–1,050 cm^−1^). Significant differences were observed in the bands of *M. wesenbergii* before and after the experiment, such as the polysaccharides (3,700–3,000 cm^−1^), C–H characteristic peak (3,000–2,800 cm^−1^), and the glycosidic bond (835 cm^−1^). Strong peaks near 3,400–3,500 cm^−1^ bands were also observed, which belong to the stretch vibrations of N–H and O–H (Zongqian et al., 2019). These peaks were likely due to the presence of carboxylic acids, alcohols, and phenolic compounds (Mecozzi et al., 2001; Badiaa et al., 2010). The broad peak of 3,408–3,431 cm^−1^ in the spectrum of *M. aeruginosa* was stronger than that before the experiment, and the intensity of the spectrum of *M. wesenbergii* was stronger. This peak was attributed to hydrogen bonds such as C–H, N–H, and O–H (Zongqian et al., 2019), indicating that there were compounds with the structure of R–NH_2_ and R–CO–NH_2_. The C═C stretching vibration at 1,657 cm^−1^ was enhanced, and the C═C carboxylic acid (C═C skeleton vibration) at 1,367–1,384 cm^−1^ was obvious. The peak at 836–1,081 cm^−1^ represents the carbon–hydrogen bonds of polysaccharides that were converted from glycosides at 836 cm^−1^ to polysaccharides (Christopher et al., 2011). The sharp increase in the intensity of the peak at 1,081 cm^−1^ also indicates that polysaccharides are one of the important factors in the formation of surface blooms.

## 4 Discussion

Our study reveals a novel mechanism for the rapid flotation of *Microcystis* under light conditions, which is species-specific. Under such conditions, *M. wesenbergii* was capable of forming surface scum, while *M. aeruginosa* could not form visible scum throughout the experiments. This timescale is shorter than the observed timescale for scum formation in conventional bloom formation process. The timescale for the formation of cyanobacterial blooms in the wild is influenced by various factors, including the nutrient status of the water body, temperature, and light intensity. Generally, the formation of cyanobacterial blooms can range from several days to several weeks. In highly eutrophic waters, under warm and stable climatic conditions, blooms may develop rapidly (Christopher et al., 2011). Considering the difference in DO and EPS content between the treatments in our study, we speculated two possible reasons for the observed phenomenon: 1) exopolysaccharides may play an important role in algal cell adhesion and surface bloom formed, and 2) the rising process of O_2_ bubbles accompanied by algal migration to the surface improves the biological amount leading to the formation of surface blooms. Under strong light conditions, the formation of bubbles can drive the formation of surface blooms.

For the first hypothesis, we analyzed the extracellular substances secreted by two *Microcystis* species. Light could affect the extracellular substances secreted by *Microcystis* and produce varying amounts of polysaccharides, thus affecting the aggregate formation. The SEM results confirmed the presence of significant secretions in the *M. wesenbergii* samples. These secretions can promote aggregate formation and upward migration to the water surface with the help of the micro-bubbles (**Figure 1D**). The S-EPS released from *M. wesenbergii* solution played an important role in the aggregation process (**Figures 3A, B**).

Studies have shown that proteins and polysaccharides are indispensable for maintaining the cross-linked structure of extracellular substances (Yang et al., 2008; Gan et al., 2012; Huiqun et al., 2018). The effect of EPS on the microbial aggregation process is mainly reflected in the bridging effect. However, the alteration of different types of EPS or the proportion of certain components can also affect this process. Meanwhile, the protein can promote the stability of maintaining the polymer structure (Badiaa et al., 2010). The protein in EPS contains more negatively charged amino acids (**Figure 2**). Compared with polysaccharides, the electrostatic bridging between EPS and divalent and polyvalent cations is more obvious. The existence of EPS and algal aggregates also contributes to better preservation of the integrity of colony algal cells. Furthermore, B-EPS, which tightly binds to the cell membrane of the algae, can serve as a binding molecule between algae cells, promoting the combination of *Microcystis* cells into a colony structure. CLSM results also confirmed that the large algal floc that rises to the surface was formed by large amounts of proteins and polysaccharides that adhere together tightly to form a “macroalgal collective” (**Figure 1B**). Indeed, EPS with multifunctional groups can promote the adsorption and chelation with various organic and inorganic matter to form aggregates in aqueous systems (Bai et al., 2016; Zhang et al., 2019).

The FT-IR spectroscopy of the *Microcystis* floating and migrating to the surface formed the surface film (**Figure 6**). This provides physical and chemical evidence for its formation and helps understand the occurrence of possible biomolecular groups or chemical changes. During the formation of surface blooms, the main functional groups in the extracellular substances secreted by *Microcystis*, such as hydroxyl and carboxyl groups, could have acted as binding sites and bridges with the high-molecular-weight adsorption sites of *Microcystis* S-EPS to form a network structure, which tightly packed single-celled *Microcystis*, and bubbles rise with photosynthesis (**Figure 1**). FT-IR analysis results showed that all samples contained a large number of hydrogen bonds, as evidenced by the broad peak of 3,500–3,400 cm^−1^ and 3,431–3,408 cm^−1^. There were noticeable changes in glycosidic bonds. The EPS was generally supported by a hydrogen bond system, and a higher ratio of hydrogen bonds resulted in stronger intermolecular interaction (Zhu et al., 2014). These findings were consistent with those of previous studies (Yang et al., 2016; Li et al., 2021).

For the second hypothesis, we analyzed the changes in oxygen quantity and electric potential during the formation of surface blooms. By modulating their buoyancy using internal gas vesicles, individual *Microcystis* can migrate along the water column at a speed of up to 1 mm/h (Walsby and Anthony, 1994). At the same time, the bubbles formed by the increase in sample oxygen are beneficial to the formation of water blooms on the surface of *Microcystis* (Johnk et al., 2008). This supports our conclusion that the rising process of O_2_ bubbles is accompanied by algal migration to the surface, which improves the biological amount and forms surface blooms. The O_2_ supersaturation is generally caused by concentrated photosynthesis, which in turn forms bubbles. The trapped O_2_ bubbles provide lift and gather most of the biomass on the surface of the water column to form a denser foam layer. Also, this rapid migration process is irreversible. The foam will remain stable for several weeks if the nutritional conditions are sufficient. According to literature reports, blooming occurs without a major increase in overall biomass, and the threshold for irreversible migration concentration is above 10^6^ cells/mL (Klemer et al., 1982). As cyanobacteria migrate toward the free surface, their effective concentration increases, and the system moves away from the critical point for blooming. The rate of oxygen production per unit biomass decreases under low light intensity or under weak photosynthetic mechanisms in harsh environments (Dervaux et al., 2015). Instead, light boosts photosynthesis and makes more bubbles. It is possible that oxygen production is the limiting factor for bloom formation.

The zeta potential is the potential of the shear plane in the double electric layer of charged particles in solution, which can reflect the stability of the colloidal system (Zhao et al., 2017). During the experimental stage (**Figure 2B**), the main force in *M. wesenbergii* solution is gravity rather than repulsion. The surface charge of protein molecules in the system is high, which maintains the stability of the system through electrostatic repulsion, making it difficult for protein molecules to accumulate (Bojorquez-Velazquez et al., 2016).

In addition, we performed a Fisher exact test bar plot on the species richness of *M. wesenbergii* before and after the experiment (**Supplementary Figure 2**). The results indicated a significant increase in species abundance at the level of *Microcystis* phylum after the experiment. Subsequently, individuals were randomly sampled from the specimens, and the dilution curve was based on the number of individuals and species (**Supplementary Figure 3A**). The species richness of *M. wesenbergii* before and after the experiment was compared by drawing dilution curves. Under the condition of extracting the same sequence, the number of operational taxonomic units (OTUs) of *M. wesenbergii* was higher after the experiment, indicating that the species richness of *M. wesenbergii* was higher. In this dilution graph (**Supplementary Figure 3B**), eventually, the curve tended to flatten out, further indicating that the number of samples taken was reasonable. Alpha diversity refers to the diversity within a specific region or ecosystem. The statistical t-test was used to detect significant differences between each of the two sets of index values (**Supplementary Figure 3C**).

Environmental isolates of *M. wesenbergii* form large colonies and exhibit complex vertical migration dynamics due to their dynamic glycan ballast ability to compete with oxygen-mediated upward migration. While similar experiments on indoor samples may still be qualitative, it would be interesting to test the mechanisms identified in this study using natural samples. Although the aggregation and binding of cyanobacteria EPS to form algal blooms have been reported (Parker et al., 2000; Micheletti et al., 2008), the underlying mechanisms were still poorly understood, and more work is needed to elucidate these binding processes. In this regard, similar experiments on algae with differences in EPS production can provide valuable data for further understanding the cell aggregation process. In future studies, we should also focus on effective and special substances that facilitate the aggregation process and identify the structure and properties of these substances. The dominant niche of high temperature-adapted cyanobacteria genera will be further reinforced with global warming and elevated carbon dioxide in the future. The cyanobacterial dominance and succession are inherently attributed to the distinctive traits of cyanobacteria including colony formation, gas vesicles, toxin release, and nitrogen fixation (Wang et al., 2021; Firsova et al., 2023). In the future, we will further explore the extracellular substances released by *Microcystis* and the physiological and biochemical effects of *Microcystis*.

## 5 Conclusion

The present study revealed that the two different species of *Microcystis* (*M. wesenbergii* and *M. aeruginosa*) ascended and gathered under light and dark conditions. It focused on the impact of the EPS and bubbles released by *Microcystis* on the formation of surface scum during the process of floating and gathering to the surface scum. The results showed the following:

(a) EPS produced by *M. wesenbergii* can contribute to the formation of large aggregates.
(b) The formation of aggregate and micro-bubble can drive the surface scum formation.
(c) Light and EPS contributed to the formation of the large algal aggregate.

## Data availability statement

The original contributions presented in the study are included in the article/**Supplementary Material**. Further inquiries can be directed to the corresponding author.

## Author contributions

TY: Conceptualization, Data curation, Formal Analysis, Validation, Visualization, Writing – original draft, Writing – review & editing. JP: Data curation, Methodology, Project administration, Visualization, Writing – review & editing. HW: Conceptualization, Writing – review & editing. CT: Supervision, Project administration, Resources, Writing – review & editing. CW: Supervision, Project administration, Resources, Writing – review & editing. BX: Supervision, Project administration, Resources, Writing – review & editing. MP: Supervision, Project administration, Resources, Writing – review & editing. XW: Funding acquisition, Project administration, Resources, Software, Supervision, Writing – review & editing.
