# Supplementary material for: Rapid flotation of Microcystis wesenbergii mediated by high light exposure: implications for surface scum formation and cyanobacterial species succession
Source: Front Plant Sci. 2024 Apr 3;15:1367680. doi: 10.3389/fpls.2024.1367680 (PMC11022887; doi:10.3389/fpls.2024.1367680)
Supplement: Supplementary file 1 [file DataSheet_1.docx]

**Supplementary Material for**

**Rapid flotation of *Microcystis wesenbergii* mediated by high light exposure: implications for surface scum formation and cyanobacterial species succession**

**TiantianYang^1,2^, Jiaxing Pan^1,3^, Huaming Wu^4^, Cuicui Tian^1,2^, Chunbo Wang^1,2^, Bangding Xiao^1,2^, Min Pan^2^, Xingqiang Wu^1*,2^**

*^1^Key Laboratory of Algal Biology of Chinese Academy of Sciences, Institute of Hydrobiology, Chinese Academy of Sciences, Wuhan 430072, China*

*^2^Kunming Dianchi & Plateau Lakes Institute, Dianchi Lake Ecosystem Observation and Research Station of Yunnan Province, Kunming 650228, China*

*^3^China Three Gorges University, Yichang,443002, China*

*^4^Institute for Environmental Sciences, University of Koblenz-Landau, Landau 76829, Germany*

^*^Corresponding author

E-mail address:Xingqiang Wu (xqwu@ihb.ac.cn)

**Figure**

**
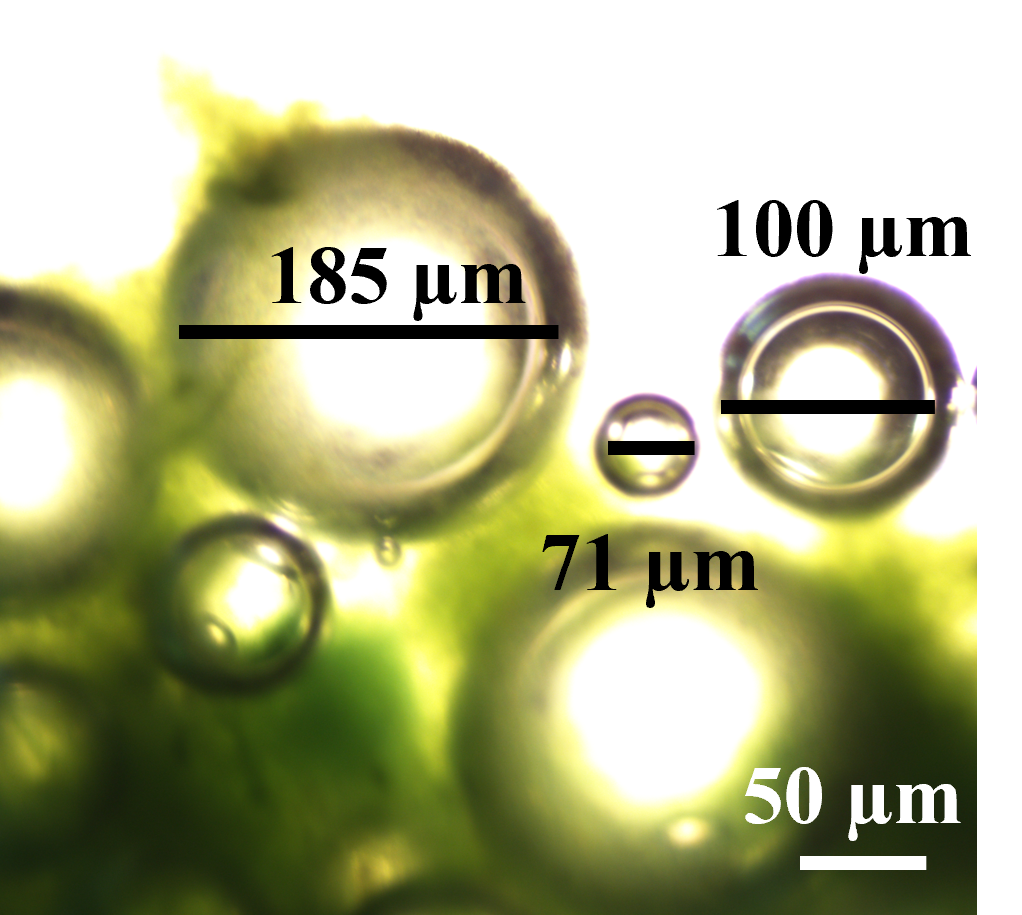
**

**Fig. S1** The size of the adhered bubbles after the bloom on the surface of *Microcystis* *wesenbergii* is generated


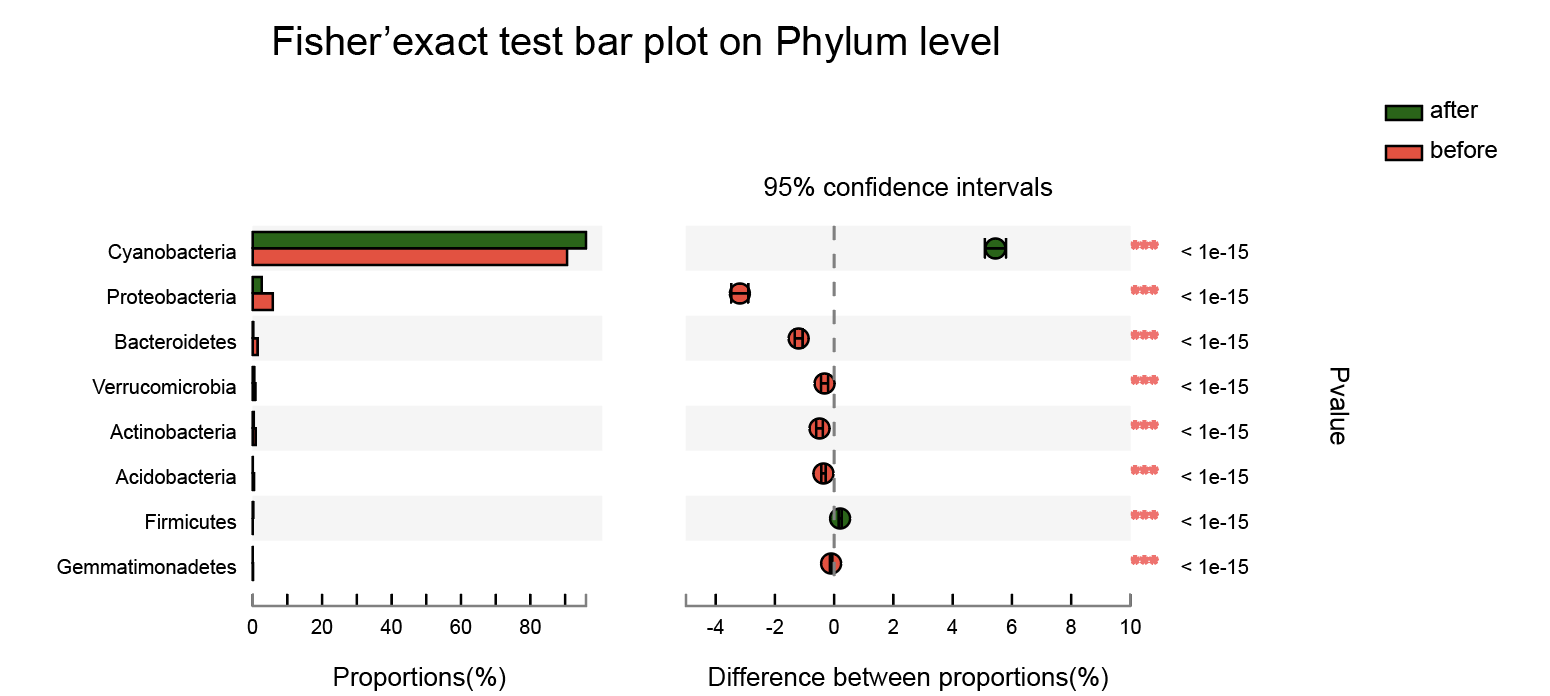


**Fig.S2** Fisher exact test bar plot on phylum level of M. wesenbergii before and after the experiment (The Y axis represents the species name at a certain classification level, and each column corresponding to the species represents the relative abundance of the species in each sample, and different colors represent different samples. The dot color shows the difference of relative abundance of species in two samples, the I interval on the dot is the upper and lower limit of the difference, and the right is the P value.)


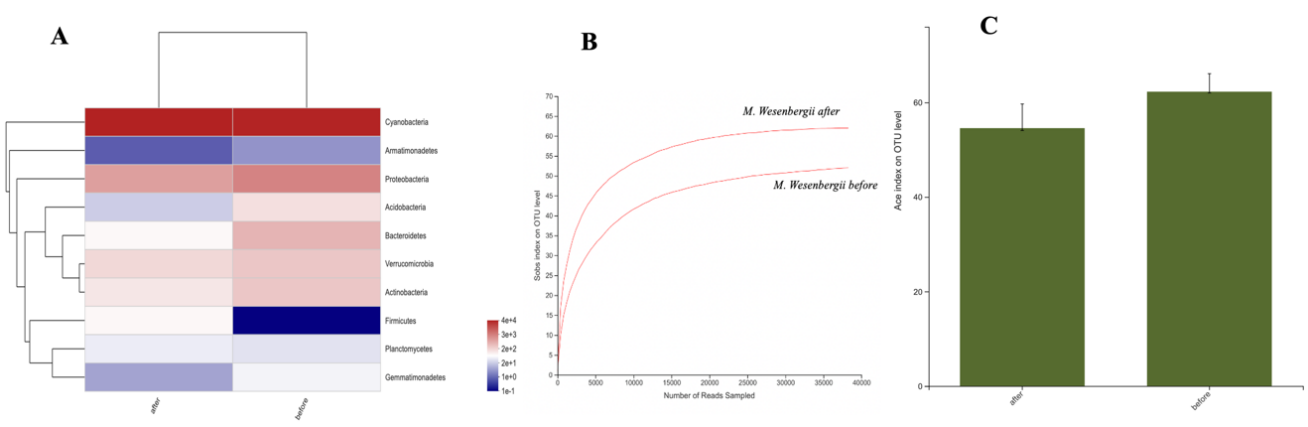


**Fig.S3** Microbial community structure analysis (A: Community heatmap analysis on phylum level; B: Alpha diversity dilution curve; C: Ace index on OUT level)

**Table S1** The composition of the stock solution of BG11 medium (100X, Blue-Green Medium)

|  | **[mg L^−1^]** |
| --- | --- |
| NaNO_3_ (sodium nitrate) | 1500 |
| K_2_HPO_4_ 3H_2_O (dipotassium phosphate) | 31.4 |
| MgSO_4_ .7H_2_O (magnesium sulfate) | 36.0 |
| CaCl_2_.2 H_2_O (calcium chloride) | 36.7 |
| citric acid | 5.6 |
| Ferric ammonium citrate | 6.0 |
| EDTA (dinatrium-salt) | 1.0 |
| Na_2_CO_3_ (sodium carbonate) | 20.0 |
